# Next-generation nano-biocatalysts: extremozyme-based nanoparticle system for cypermethrin bioremediation

**DOI:** 10.1007/s00253-026-14053-z

**Published:** 2026-10-17

**Authors:** Juhi Barot, Nafisa Patel, Vimal Prajapati, Nadiah S. Alzharani, Andrea Mastinu

**Affiliations:** 1 Naran Lala College of Professional and Applied Sciences, affiliated to Veer Narmad South Gujarat University, Navsari, India; 2 Aspee Shakilam, Biotechnology Institute, Navsari Agriculture University, Surat, Gujarat India; 3 Department of Public Health, College of Applied Medical Sciences, Al Baha University, Al Baha, 65779 Saudi Arabia; 4 Department of Molecular and Translational Medicine, Division of Pharmacology, University of Brescia, Brescia, 25123 Italy

**Keywords:** Biogenic selenium nanoparticles, Bioremediation, CCD-RSM, Cypermethrin, Extremozyme, Laccase, PBD

## Abstract

**Abstract:**

Extremozymes, with their exceptional catalytic activity and stability under extreme environmental conditions, have great potential for pesticide remediation. This study involves the isolation and screening of a new extremozyme-producing microbe, *Proteus mirabilis* JB1, for the production of alkaline-pH-stable laccase. A primary OFAT screening tested a broad range of carbon sources (glucose, lactose, xylose, sucrose, starch, glycerol, cellulose, trisodium citrate, sodium acetate), nitrogen sources (ammonium sulfate, sodium nitrate, urea, beef extract, peptone, casein, gelatin), salts (0.1 mM and 0.01 mM) such as CuSO₄, KCl, ZnCl₂, MgCl₂, MnCl₂, NiCl₂, NaCl, and CaCl₂, and pH levels (7–10). The highest enzyme activity of 0.999 U/ml was achieved using 0.25% (w/v) cellulose, 0.8% (w/v) urea, 0.1 mM MnCl_2_, MgSO_4_, CaCl_2_, NaCl, and KCl, and 0.01 mM CuSO_4_, FeSO_4_, and NiCl_2_ at pH 9 with good stability to surfactants (Tween 80, SDS), organic solvents (acetone, ethanol, methanol), and inhibitors (EDTA). PBD and CCD were then implemented to screen and optimize critical parameters—cellulose (1.5% w/v), urea (0.7% w/v), and MgSO₄ (0.05 mM)—to achieve an efficient increase in enzyme activity to 2.3 U/mL, demonstrating the strength and efficacy of the optimization process. Biogenic selenium nanoparticles (Bio-SeNPs) were fabricated and exhibited very high efficiency in pesticide degradation, achieving 100% degradation of cypermethrin without forming any toxic intermediates within 72 h. This research has provided a comprehensive and replicable technique covering microbial isolation, enzyme optimization, fabrication of a nanobiocatalyst, and its application.

**Key points:**

*• A novel isolate, Proteus mirabilis, was employed for the production of the extremozyme laccase.*

*• Optimization increased the yield by 9.58-fold.*

*• Bio-SeNPs were employed to degrade cypermethrin, achieving 100% detoxification within 72 h.*

**Supplementary Information:**

The online version contains supplementary material available at https://doi.org/10.1007/s00253-026-14053-z.

## Introduction

Cypermethrin, a synthetic pyrethroid insecticide, is widely employed in agricultural and domestic pest control due to its high efficacy and broad-spectrum activity. However, its extensive use has led to significant environmental accumulation, particularly in soil and aquatic ecosystems (Poli and Motireddy 2025; Rehman et al. 2024). Cypermethrin exhibits high persistence, low water solubility, and strong affinity for organic matter, which contributes to its bioaccumulation and toxicity in non-target organisms. Chronic exposure has been linked to neurotoxicity, endocrine disruption, and ecological imbalance, prompting urgent efforts to develop sustainable remediation strategies (Kansal et al. 2023).

Bioremediation, which leverages the catalytic potential of microbial enzymes, offers a promising alternative to conventional physicochemical methods, which often entail high energy demands, incomplete degradation, and secondary pollution (Kaur et al. 2024; Tarfeen et al. 2022). The development of cost-effective, robust, and sustainable strategies to remediate the recalcitrant pollutants requires diversifying from the available biocatalysts. Many fungal species, such as *Aspergillus niger* and *Trichoderma harzianum*, are well documented for their ligninolytic and cellulolytic enzyme profiles (Abdusamatov et al. 2026); the potential of bacterial extremozymes remains an emerging area for sustainable bioremediation. Among the enzymes explored for pesticide degradation, laccase (EC 1.10.3.2) stands out for its broad substrate specificity, ability to oxidize a wide range of phenolic and non-phenolic compounds, and minimal cofactor requirements. Laccases have effectively degraded various environmental pollutants, including dyes, pharmaceuticals, and pesticides, making them attractive candidates for cypermethrin detoxification (Orlando et al. 2024; Rajendran et al. 2025). Despite their catalytic versatility, free laccase enzymes often exhibit limited operational stability under environmental conditions, including fluctuating pH and temperature, as well as the presence of organic solvents. To overcome the stability limitation, nanobiocatalyst systems have emerged as a powerful strategy to enhance enzyme performance (Fahim et al. 2025; Tan et al. 2023). Selenium nanoparticles (SeNPs), in particular, have attracted attention for their biocompatibility, antioxidant properties, and ability to interact with biomolecules (Garza-García et al. 2023; Raturi et al. 2025). SeNPs offer a green and scalable platform for enzyme immobilization when synthesized via biological routes (Bisht et al. 2022; Ibrahim et al. 2024).

Studies suggest that selenium nanoparticles can synergistically enhance the enzyme's catalytic activity due to their stability and surface functionality (Bisht et al. 2022). This hybrid system enhances enzyme stability and reusability while also improving substrate accessibility and electron transfer during degradation reactions (Ren et al. 2024; Xu et al. 2023). Moreover, the nanoscale interface facilitates better dispersion in contaminated matrices, thereby increasing the contact efficiency with hydrophobic pollutants, such as cypermethrin (Asghar et al. 2024). Although SeNPs are very promising in bioremediation, there are fewer reports on integrating microbial laccase production, SeNP generation, enzyme immobilization, and pesticide degradation. The ability of Lac@SeNPs to completely detoxify pesticides remains unknown.

The current study attempts to fill the research gap by integrating the synergistic activity of laccase production with selenium nanoparticles (Lac@SeNPs), as bioactive functional nanocarriers for specific environmental applications. The study aims to isolate, screen, and characterize a microbial strain from the extreme environment of industrial effluent, distinct from the commonly studied fungal laccases and well-characterized *Bacillus* strains. The isolate shows an exceptional adaptation to tolerate extreme alkaline conditions, coupled with enzyme stability and retained activity at elevated pH. Optimization using statistical approaches includes one-factor-at-a-time (OFAT), Plackett–Burman design (PBD), and Central Composite Design (CCD) to enhance enzyme activity and subsequent enzyme immobilization. The majority of the literature reports on conventional immobilization supports, such as inert materials like chitosan or silica-based materials, with few reports on magnetic iron oxide (Fe₃O₄). The use of Selenium Nanoparticles (SeNPs) as a carrier offers distinct advantages, including high surface area and high catalytic efficiency. The biologically synthesized selenium nanoparticles are conjugated with purified laccase to form Lac@SeNPs and evaluated for degradation efficiency against cypermethrin under controlled conditions. This integrated approach aims to develop a stable and eco-friendly nanobiocatalyst for the effective remediation of cypermethrin-contaminated environments. Leveraging the unique metabolic pathways of these novel bacterial strains may become a potential tool to address the complex challenges associated with pesticide decontamination.

## Materials and methods

### Sample collection and preparation

Environmental samples were collected from five locations known for industrial and organic contamination to isolate potential laccase-producing microorganisms. The sampling sites included: Industrial effluent discharge points; biological sludge from wastewater treatment plants; Chemical sludge from industrial processing units; and industrially contaminated soil zones (21.099795416306733, 72.98735113010757); and agro waste composting soil (20.924622842084553, 72.90838138566238). Approximately 100–200 g of solid samples and 100 mL of liquid effluent were collected in sterile containers, transported on ice, and processed within 24 h (Oyedeji et al. 2025). The potential isolate, *Proteus mirabilis* JB1, was isolated from industrial chemical sludge and maintained at the microbiology department of Naran Lala College of Professional and Applied Sciences, Navsari, India. In addition, the 16S rRNA sequence is been deposited in GenBank under accession number PQ160485 and will be available from the corresponding author upon request for research purposes.

### Enrichment and primary screening for laccase-producing microbial strains

10 mL of each sample (10 g of soil/10 mL of sludge) was inoculated into 100 mL of Minimal Salt Medium (MSM) supplemented with 2 mM guaiacol as an indicator substrate and 0.5% (w/v) glucose as a carbon source to enrich for laccase-producing microorganisms. The medium composition was as follows: K_2_HPO_4_ (0.173% w/v)_,_ KH_2_PO_4_ (0.0680% w/v), MgSO_4_.7H_2_O (0.01% w/v), NaCl (0.4% w/v), FeSO_4_.7H_2_O (0.003% w/v), CaCl_2_.2H_2_O (0.002% w/v), CuSO_4_ 0.2 mM (Bibi et al. 2023). Cultures were incubated at 37 ± 1 °C for 72 h under shaking conditions (150 rpm). After enrichment, isolation was performed from serially diluted samples (10^−1 to 10^−6). 0.1 ml of each dilution was plated on guaiacol agar plates (MSM + 1.5% agar + 0.2 mM guaiacol) and incubated for 48–72 h. Colonies exhibiting reddish-brown halos around their growth on guaiacol-containing agar were considered presumptive laccase producers, as laccase oxidizes guaiacol to a polymer (Alshammary et al. 2025).

### Secondary screening and quantitative laccase assay

Purified isolates were cultured in liquid MSM at pH 7 and 9, supplemented with 0.2 mM guaiacol, and incubated under identical conditions. After 120 h, cultures were centrifuged at 10,000 × g for 10 min at 4 °C, and the supernatant was used for enzyme assays. Laccase activity was determined at room temperature using guaiacol as a substrate. The reaction was performed in a 100 mM sodium acetate buffer (pH 5.0) containing 10 mM guaiacol. The reaction mixture consisted of 3.0 mL of buffer, 1.0 mL of guaiacol solution, and 1.0 mL of crude culture supernatant from the enzyme source, resulting in a final volume of 5.0 mL. The mixture was incubated for 10 min at room temperature in the dark and under static conditions, after which the absorbance was measured at 470 nm using a UV spectrophotometer (UV-1900, Shimadzu)(Vantamuri and Kaliwal 2016).

One unit (U/mL) of laccase activity was defined as the amount of enzyme required to oxidize 1 µmol of guaiacol per minute under the assay conditions. Enzyme activity was calculated using the following equation:

$$Enzyme \;Activity \;(U/mL) =\frac{ \left(Ab{s}_{470}\times 4 \times {V}_{t}\times Dilution \;Factor\right)}{\left(\epsilon \times {V}_{s}\right)}$$

where:

Vt: 5.0 mL → final volume of the reaction.
Vs: 1.0 mL → enzyme sample volume.
ε: 6740 M^−1^ cm^−1^ → extinction coefficient of guaiacol.

### Molecular identification of a potential isolate

The best laccase producer was characterized morphologically and biochemically, and further identification was carried out by 16 s rDNA gene, for which the genomic DNA of the potential bacterial isolates was extracted, and the 16 s rDNA gene was amplified using the universal primers (F, 5’ AGAGTTTGATCCTGGCTCAG 3’; R, 5’TACGGYTACCTTGTTACTT 3’) (Frank et al. 2008). Amplification was carried out in a thermal cycler (Applied Biosystems Veriti) using the following reaction profile: initial denaturation at 95 °C for 3 min, followed by 35 cycles of denaturation at 95 °C for 30 s, annealing at 55 °C for 30 s, and extension at 72 °C for 01 min, with a final extension at 72 °C for 5 min. The purified PCR product was sequenced, and the phylogenetic relationship of the isolate was determined by comparing the obtained sequence with sequences available in the GenBank database of the National Center for Biotechnology Information (NCBI) (Weisburg et al. 1991).

### Media optimization by one-factor-at-a-time (OFAT)

A One-Factor-at-a-Time (OFAT) approach was employed to optimize laccase production by systematically evaluating individual media components and culture conditions. Three basal media—Luria–Bertani, Nutrient Broth, and Minimal Medium—were compared, with Minimal Medium selected for its superior enzyme yield (Nandal et al. 2013). The best basal medium, which yielded good enzyme activity, was chosen for further analysis. The selected basal medium was supplemented with 1% w/v carbon sources (fructose, galactose, maltose, lactose, xylose, sucrose, dextrose, starch, glycerol, tri-sodium citrate, sodium acetate, cellulose). Different organic and inorganic nitrogen sources at a concentration of 0.2% w/v (yeast extract, ammonium sulfate, sodium nitrate, urea, peptone, beef extract, casein, gelatin) were tested to identify the most supportive substrates for laccase induction. Among these, the best carbon and nitrogen sources were tested at different concentrations of 0.25, 0.50, 1.00, 2.00, 4.00, and 8.00% w/v, and 0.05, 0.10, 0.20, 0.40, 0.80, and 1.60% w/v, respectively(Arumugam and Uthandi 2024; El-Bendary et al. 2021). Nine metal ions (CuSO_4_, FeCl_3_, KCl, ZnCl_2_, MgCl_2_, MnCl_2_, NiCl_2_, NaCl, CaCl_2_) were tested at 0.01 mM and 0.1 mM concentrations to assess the role of trace elements. Furthermore, the influence of pH was studied using five buffer systems with a concentration of 10 mM: Tris–HCl (pH 7.0, 8.0, 9.0, 10.0), phosphate (pH 7.0), carbonate–bicarbonate (pH 9.0 and 10.0), Tris-citrate (pH 8.0), and sodium acetate (pH 7.0). The tolerance and enzyme activity production of the strain under the effect of surfactants at concentrations of 0.1 mM, 1 mM, and 5 mM (Tween 80, SDS), inhibitor (EDTA), and organic solvents at concentrations of 5% and 20% (acetone, ethanol, methanol) were checked (Britos et al. 2018).

### Media optimization by plackett–burman design (PBD)

The Plackett–Burman (PB) design is a powerful technique for screening and evaluating variables that affect enzyme production (Plackett and Burman 1946). In the present study, seven factors, including cellulose, sucrose, urea, beef extract, peptone, and MnCl_2_ and MgSO_4_, were screened using a PB design to identify the significant factors affecting laccase enzyme production (Table 1). The experimental design for screening variables is presented in Table 2. All variables were treated as numerical factors and explored at two widely spaced levels: low and high. The following equation calculated the individual parameters of laccase production:

$$E\left(Xi\right)=2\left(\Sigma {M}^{+}-\Sigma {M}^{-}\right)/N$$

where E is the effect of the parameter under study on Laccase production at which the parameter was at its highest and lowest, respectively, and N is the total number of trials.

**Table 1:** Components and their values used in Plackett- Burman design for laccase production using *Proteus mirabilis* JB1

	Component	Low Value	High Value
1	Cellulose	0.1%	1.2 g%
2	Sucrose	0.1%	1.5 g%
3	Urea	0.05 g%	2 g%
4	Beef Extract	0.05 g%	2 g%
5	Peptone	0.05 g%	2 g%
6	MnCl2	0.01 mM	0.1 mM
7	MgSO4	0.01 mM	0.1 mM

**Table 2:** Plackett–Burman design generated by the fractional rotation of a full factorial design, where X1–X7 are independent variables and D1–D2-D3-D4 are dummy variables

		X1	X2	X3	X4	X5	X6	X7	X8	X9	X10	X11
Run No	Laccase Activity (U/ml)	Cellulose	Sucrose	Urea	Beef Extract	Peptone	MnCl2	MgSO4	D1	D2	D3	D4
1	1	High	High	low	High	High	High	low	low	low	High	low
2	1.27	low	High	High	low	High	High	High	low	low	low	High
3	**1.89**	**High**	**low**	**High**	**High**	**low**	**High**	**High**	**High**	**low**	**low**	**low**
4	0.9	low	High	low	High	High	low	High	High	High	low	low
5	1.22	low	low	High	low	High	High	low	High	High	High	low
6	1.32	low	low	low	High	low	High	High	low	High	High	High
7	1.2	High	low	low	low	High	low	High	High	low	High	High
8	1	High	High	low	low	low	High	low	High	High	low	High
9	1.65	High	High	High	low	low	low	High	low	High	High	low
10	1	low	High	High	High	low	low	low	High	low	High	High
11	1.3	High	low	High	High	High	low	low	low	High	low	High
12	1.6	low	low	low	low	low	low	low	low	low	low	low

An experimental error was estimated by calculating the variation among the dummy variables as

$$Veff={\left(Ed\right)}^{2}/n$$

where *V*eff is the variance of the effect of level, Ed is the dummy variable effect, and n is the number of dummy variables used. The standard error (SE, Es) of the concentration effect was the square root of the variance of an impact, and the significance level (*P*-value) of each concentration effect was determined using Student's t-test.

$$t\left(Xi\right)=E\left(Xi\right)/Es$$

where E (Xi) is the effect of variable Xi.

A 12-run experimental matrix was generated using Design-Expert software, with randomized combinations of high and low levels for each factor. Each run was conducted in 100 mL Erlenmeyer flasks containing 50 mL of LB medium supplemented with 0.02% guaiacol and 0.2 mM CuSO₄. Cultures were incubated at 35 ± 2 °C for 72 h under steady-state conditions.

### Media optimization by central composite design (CCD)

A Central Composite Design (CCD) was employed under Response Surface Methodology (RSM) to fine-tune the concentrations of key media components identified through Plackett–Burman Design. CCD enables modeling nonlinear relationships and interactions among variables, allowing the prediction of optimal conditions for maximum laccase production (Othman et al. 2021). Based on statistical significance and biological relevance, three variables were selected for CCD: i.e., A: Urea; B: Cellulose; C: Magnesium sulfate (MgSO₄). Each variable was studied at five coded levels: − α, − 1, 0, + 1, and + α, where α = 1.68 for rotatability. The actual concentrations corresponding to these levels are shown in Table S1.

A total of 20 experimental runs were generated using Design-Expert software and were conducted in 250 mL Erlenmeyer flasks containing 100 mL of Luria–Bertani (LB) medium supplemented with 0.02% guaiacol and 0.2 mM CuSO₄. Cultures were incubated at 35 ± 2 °C for 72 h under steady-state conditions. Model adequacy was evaluated using ANOVA, R^2^, adjusted R^2^, predicted R^2^, and lack-of-fit tests. Response surface plots and contour diagrams were generated to visualize the effects and interactions among variables. The experimental design data were statistically analyzed and interpreted using Design-Expert software version 8.0 (Stat-Ease Inc., Minneapolis, MN, USA).

### Biological synthesis of selenium nanoparticles and laccase coating

Selenium nanoparticles (SeNPs) were biologically synthesized using the cell-free supernatant of the optimized laccase-producing microbial strain to enhance laccase's catalytic stability and reusability for environmental applications. Subsequently, laccase was immobilized onto the surface of SeNPs to form a nanobiocatalyst system (Lac@SeNPs), which was characterized using multiple analytical techniques.

### Immobilization of laccase onto SeNPs (Lac@SeNPs)

#### Partial purification of laccase

The microbial strain was cultivated under optimized conditions in LB medium supplemented with 0.02% guaiacol and 0.2 mM CuSO₄ to induce laccase production. After 72 h of incubation at 35 ± 2 °C and under steady-state conditions, the culture was centrifuged at 10,000 × g for 10 min at 4 °C. The resulting cell-free supernatant was filtered through a 0.22 µm membrane to ensure sterility (Kasirajan et al. 2021).

Laccase from the clarified supernatant was precipitated with ammonium sulfate to 80% saturation. The protein precipitate was dissolved in 50 mM Tris buffer (pH 8.0) and dialyzed against the same buffer at 4 °C overnight to remove residual ammonium sulfate (Illuri et al. 2021).​

#### Synthesis and immobilization of selenium nanoparticles

​Selenium nanoparticles (SeNPs) were produced using partially purified bacterial laccase as the biocatalyst. In particular, 5 mL of the enzyme solution was stirred with 30 mL of 10 mM sodium selenite. The reaction was incubated at 37⁰C for 48 h, and nanoparticle formation was observed by a noticeable color change from colorless to brick red (Abdulmumini et al. 2025).

#### Characterization of Lac@SeNPs by UV–visible spectroscopy

UV–Visible spectroscopy differentiated biologically synthesized selenium nanoparticles (SeNPs) and laccase-coated selenium nanoparticles (Lac@SeNPs). Spectral measurements were conducted on a double-beam UV–Vis. spectrophotometer (Shimadzu UV-1900) over 200–800 nm. SeNPs exhibited an inherent absorbance peak in the 250–300 nm region, confirming nanoparticle formation via surface plasmon resonance. Conversely, a minor shift and broadening of the peak occurred, along with the development of extra shoulders at 280–320 nm, which is characteristic of effective enzyme conjugation and protein–nanoparticle interaction. This spectral change facilitated the creation of a robust nanobiocatalyst system viable for further use in cypermethrin degradation (Ullah et al. 2021).

#### Characterization of laccase-coated selenium nanoparticles (Lac@SeNPs)

Fourier Transform Infrared (FTIR) spectroscopy was used to identify the functional groups responsible for stabilizing the biologically synthesized selenium nanoparticles (SeNPs) and to validate the successful immobilization of laccase on their surface. Air-dried laccase-coated SeNPs (Lac@SeNPs) were powdered and blended with spectroscopic-grade potassium bromide (KBr) at 1:100 to form translucent pellets. Spectral analysis was conducted in the range of 4000–400 cm⁻^1^ at a resolution of 4 cm⁻^1^. FTIR measurements were subcontracted to the Center of Excellence in Vapi, which has a high-resolution PerkinElmer Fourier Transform Infrared (FTIR) spectrometer for spectral acquisition (Zhao et al. 2025). Particle size distribution and zeta potential of Lac@SeNPs were measured using the NanoPlus Particle Size and Zeta Potential Analyzer (Particulate Systems, India). DLS determined the hydrodynamic diameter and PDI at 25 °C. Zeta potential was calculated using Electrophoretic Light Scattering and the Smoluchowski equation (Oliveira et al. 2026). Immobilization efficiency was evaluated by quantifying the residual protein concentration in the supernatant using a bovine serum albumin (BSA) standard curve (Figure S1). The immobilization yield was determined using the equation of Hao et al.

$$Immobilization \;yield=\frac{Total \;immobilized \;protein}{Initial \;protein}*100$$

#### Cypermethrin degradation assay

Cypermethrin degradation was assessed using three distinct biocatalytic treatments: (i) direct application of the laccase-producing microbial strain, (ii) partially purified laccase enzyme, and (iii) laccase-coated selenium nanoparticles (Lac@SeNPs). The assay was conducted in Minimal Salt Medium (MSM) supplemented with cypermethrin at concentrations ranging from 0.3 mM to 1.0 mM. For the microbial treatment, actively growing cultures were inoculated into MSM containing cypermethrin and incubated at 35 ± 2 °C under steady-state conditions. In parallel, purified laccase (10 U/mL) and Lac@SeNPs (equivalent enzyme loading) were added to separate reaction sets under identical conditions. Samples were withdrawn at defined time intervals (0, 12, 24, 48, and 72 h), and residual cypermethrin was extracted using ethyl acetate. The degradation of cypermethrin was preliminarily evaluated by monitoring changes in its characteristic absorption profile with a UV–Visible spectrophotometer over the wavelength range 200–400 nm. Alterations in the absorption characteristics (λmax) of cypermethrin were observed and further analyzed by comparing them with those of the control sample to determine the degree of pesticide degradation. A reduction or complete disappearance of absorbance at λmax indicated the removal of cypermethrin using the proposed nanobiocatalytic technique (Kansal et al. 2023). Quantification was performed via FTIR and gas chromatography (GC). For GC analysis, an electron capture detector (ECD equipped GC with an HP-5 capillary column was used under programmed temperature (initial 150 °C, ramped to 260 °C) in a carrier gas of nitrogen at 1 mL/min. Identification and quantification were performed using retention times and peak areas relative to authentic standards, with method validation including recovery rates and detection and quantification limits to enable accurate and precise analysis of cypermethrin bioremediation. (He et al. 2022).

## Result

### Primary screening and isolation of microbial strain

A total of 21 morphologically distinct microbial isolates were obtained from five different environmental samples; among these isolates, 03 were from industrial effluent, 01 from industrial biological sludge, 05 from industrial chemical sludge, 10 from agro-waste composting soil, and 02 from contaminated soil of an industrial area. All these isolates were subjected to primary screening on guaiacol-supplemented agar plates. Among these, 12 isolates exhibited reddish-brown zones indicative of extracellular laccase activity. All 12 isolates underwent secondary screening to quantify laccase production, followed by screening for potential laccase producers.

### Secondary screening and quantitative laccase activity to select potential isolate

A total of 12 morphologically distinct bacterial isolates were cultivated in liquid production medium, and extracellular laccase activity was quantified using guaiacol as the chromogenic substrate. Among these, isolate JB1 exhibited the highest laccase activity, i.e., 0.240 U/mL and 0.156 U/mL at pH 9 and pH 7, respectively, after 96 h of incubation at 37 ± 2 °C under steady state conditions. Enzymatic activity was determined by monitoring guaiacol oxidation, and results were expressed as units per millilitre (U/mL) of culture supernatant. The activity range across all isolates varied from 0.001 to 0.240 U/mL at pH 9 and 0.007 to 0.156 U/mL at pH 7, as shown in Table S2.

### Molecular identification of a potential isolate

The potential isolate JB1 was molecularly identified by 16S rDNA sequencing and compared with the NCBI database using BLASTn, showing 99% similarity to a strain with accession number OP860660. The phylogenetic tree was prepared using the neighbor-joining method (Figure S2). Based on morphological, biochemical, and molecular identification, the isolated strain was classified as *Proteus mirabilis,* and the sequence has been deposited in NCBI GenBank under accession number PQ160485.

### Optimization of laccase production using the OFAT approach

**Fig. 1:**
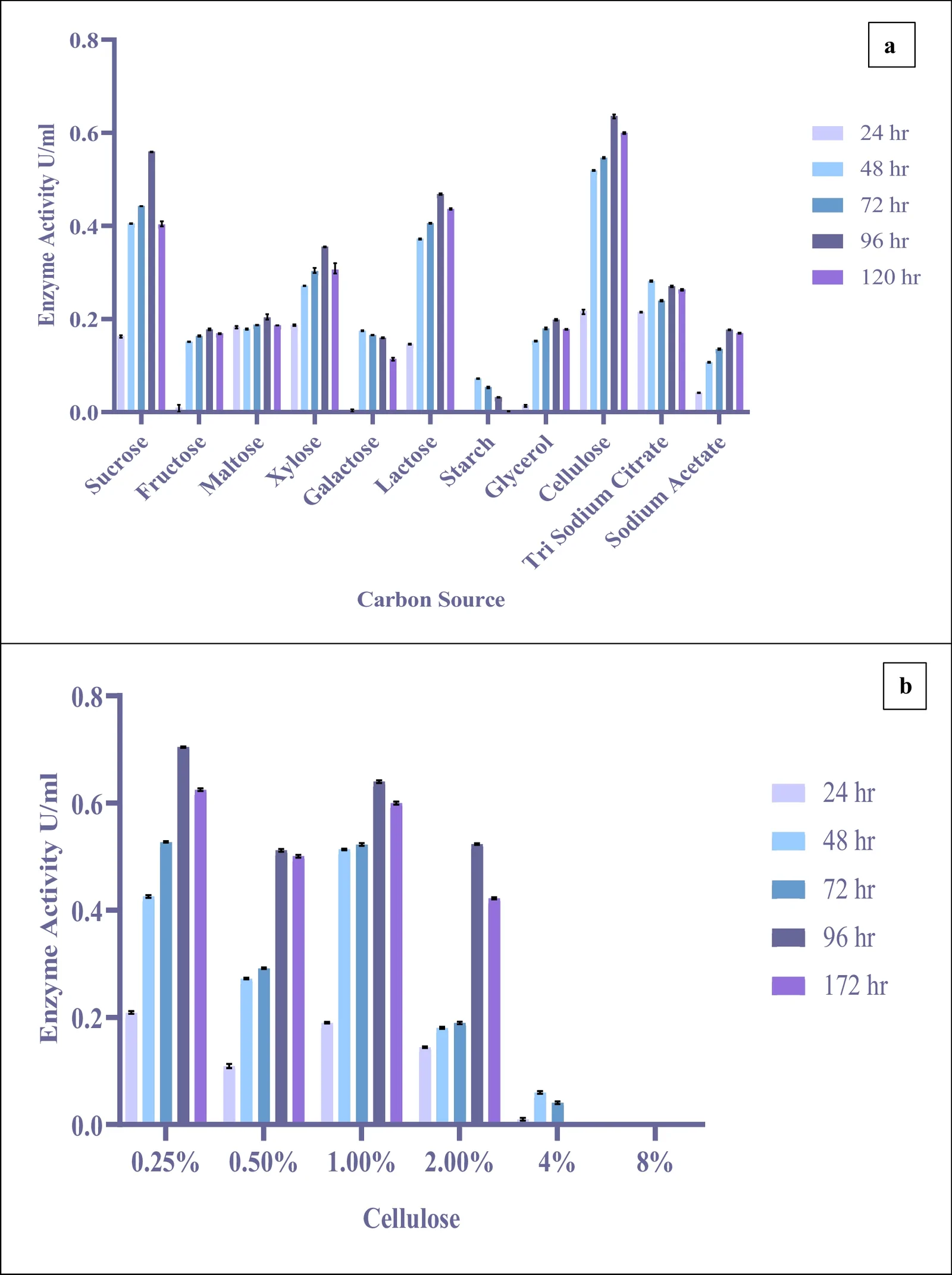

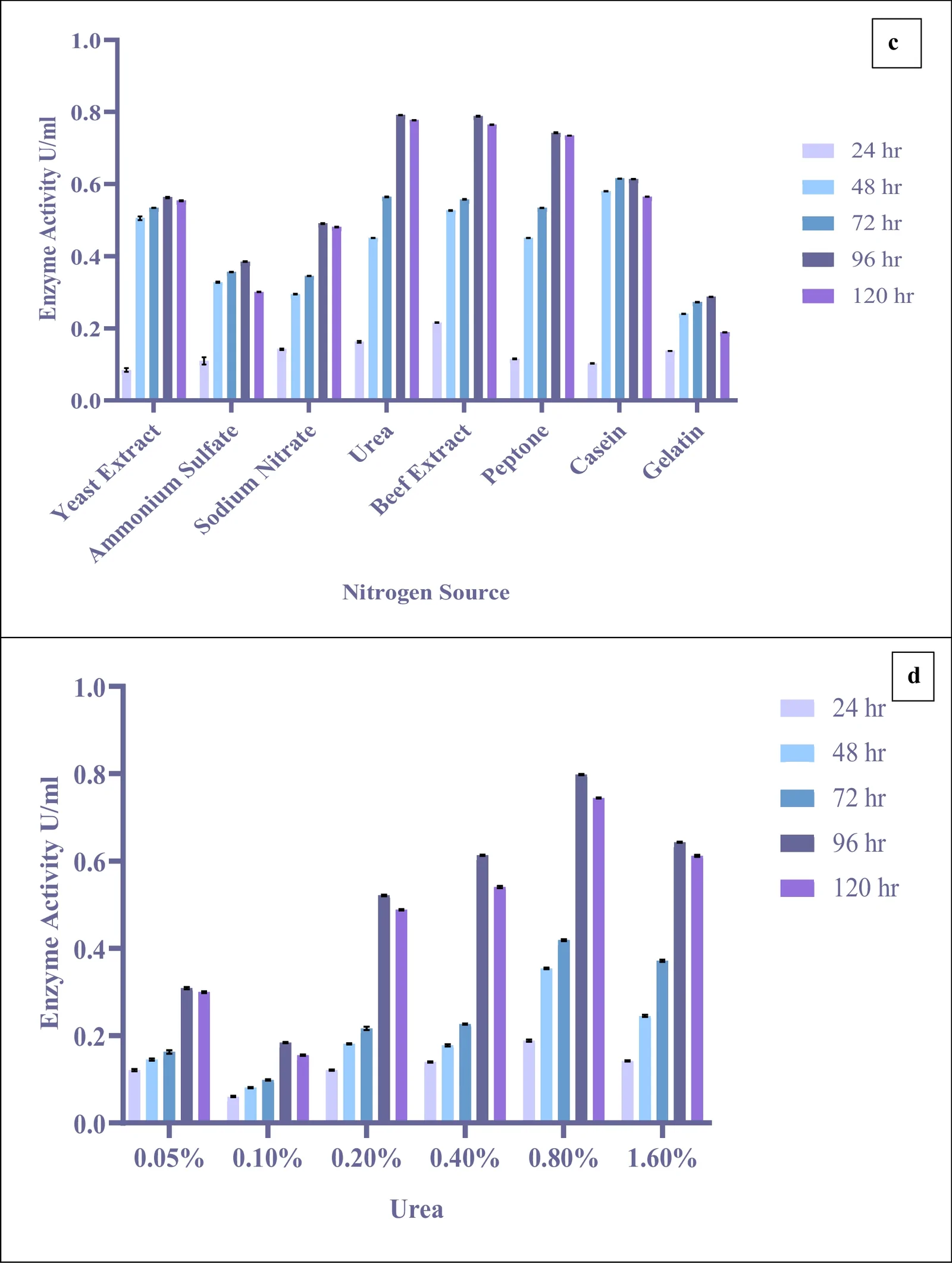

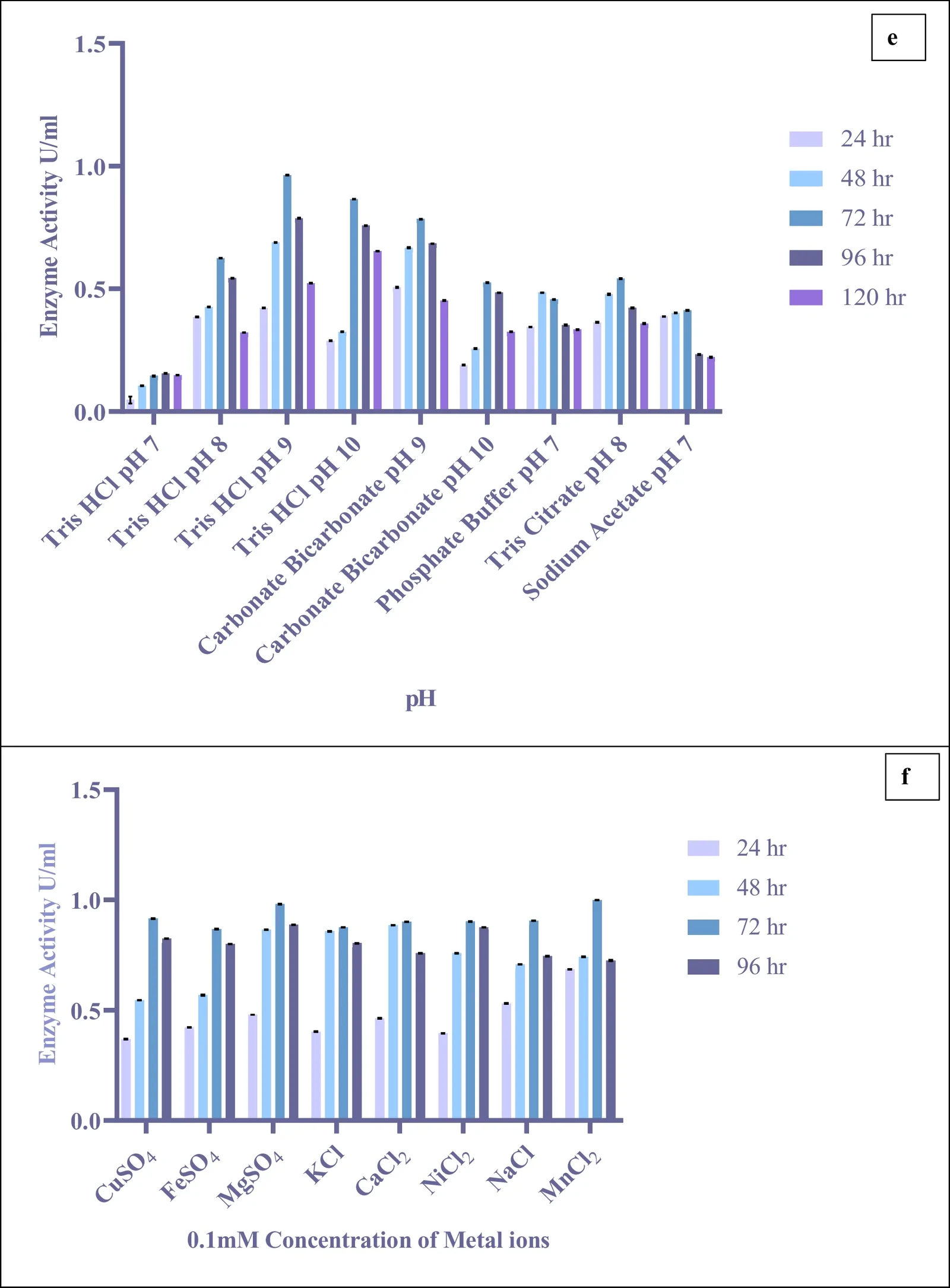

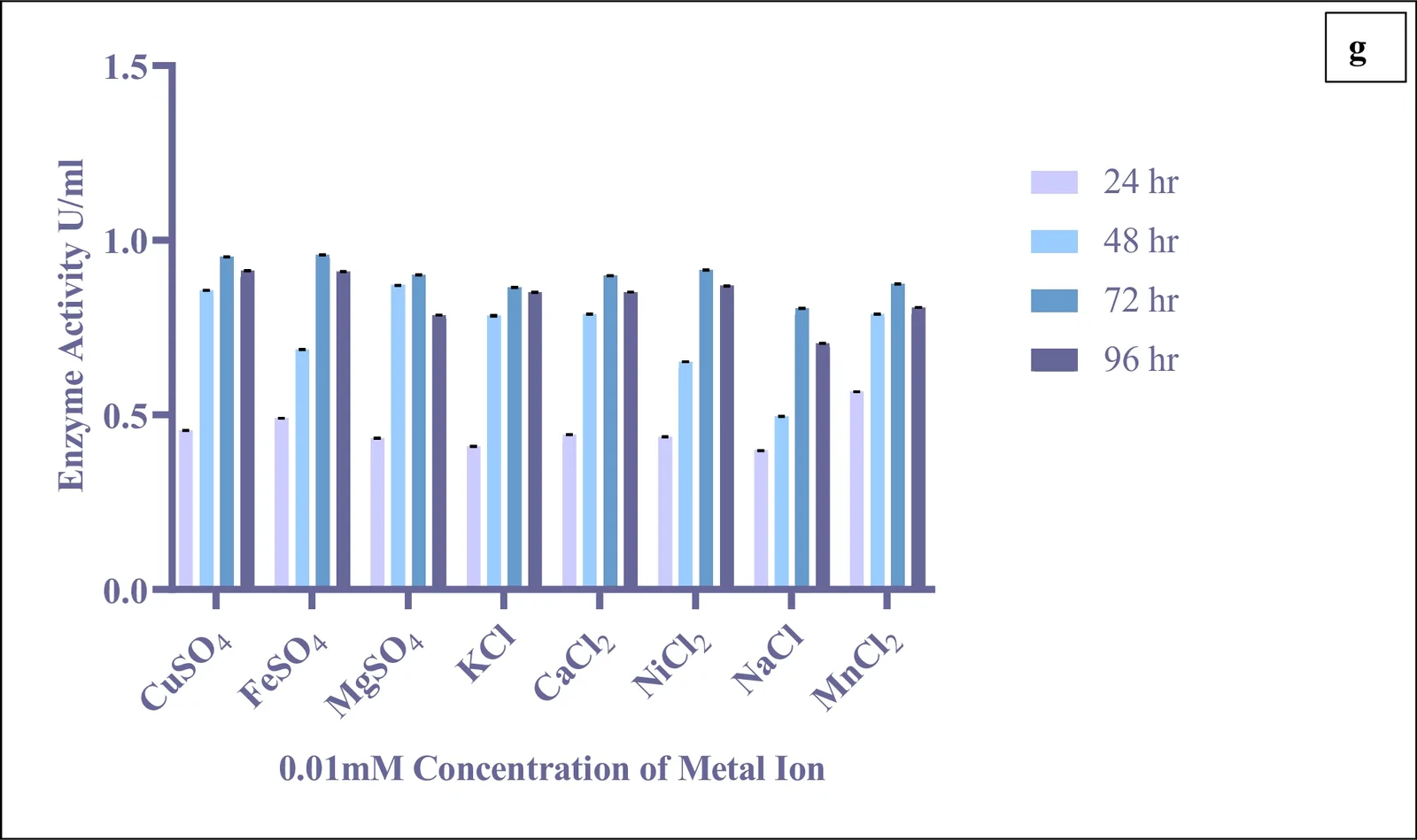
**a** Effect of various carbon sources, **b** Effect of Cellulose concentration, **c** nitrogen sources, **d** Urea concentration, **e** pH buffers, **f** metal ion concentration (0.1 mM), and (**g**) metal ion concentration (0.01 mM)

Among the three basal media used for cultivation, laccase activity was highest in LB (0.240 U/mL). Testing several carbon sources at 1% (w/v), cellulose supported the highest laccase activity (0.638 U/mL), followed by sucrose (0.558 U/mL), lactose (0.467 U/mL), and xylose (0.355 U/mL), as indicated in Fig. 1a. Further optimization of cellulose concentration revealed that only 0.25% (w/v) was required to enhance laccase production (0.705 U/ml), Fig. 1b. Among the tested nitrogen sources (0.2% w/v), organic sources, especially urea (0.792 U/mL) and beef extract (0.789 U/mL), were found to improve laccase production compared with inorganic sources such as ammonium sulfate and sodium nitrate (Fig. 1c). Concentration optimization revealed that the optimal nitrogen concentration was 0.8% w/v, resulting in an enhanced laccase activity of 0.798 U/mL (Fig. 1d). After optimization of carbon and nitrogen sources, the maximum enzyme activity was observed within 72 h. The influence of pH on laccase biosynthesis, shown in Fig. 1e, indicated the highest enzyme activity at pH 9.0 in a 10 mM Tris–HCl buffer, demonstrating that the bacterial strain can tolerate alkaline conditions, thereby improving enzyme production and secretion. Metal ion screening indicated that MnCl_2_ (0.999 U/mL), MgSO_4_ (0.981 U/mL), CaCl_2_ (0.900 U/mL), NaCl (0.905 U/mL) and KCl (0.875 U/mL) ions had the highest activity observed at 0.1 mM, whereas CuSO_4_ (0.952 U/mL), FeSO_4_ (0.958 U/mL) and NiCl_2_ (0.915 U/mL) influenced laccase activity at 0.01 mM (Fig. 1f).

The effects of surfactants, inhibitors, and organic solvents were studied to assess enzyme stability under industrially relevant conditions. For comparison, the untreated enzyme is used as the 100% activity standard. SDS was found to significantly inhibit the enzyme's activity, resulting in ~ 53–70% of the activity at all concentrations. On the other hand, the non-ionic surfactant, Tween 80, showed relatively little inhibition, with residual activity of 64–74%. The enzyme showed moderate activity (~ 44–67%) in the presence of EDTA. The enzyme activity remained relatively high in methanol and ethanol at 5% concentration, with residual activity of 81 and 65%, respectively. However, inhibition of the enzyme by acetone resulted in 20% residual activity at a concentration of 20% (v/v) (Figure S3).

### Media optimization by plackett–burman design (PBD)

A Plackett–Burman Design (PBD) was employed to statistically evaluate the influence of 07 independent variables on laccase production from OFAT analysis. Cellulose and sucrose were found to be the best carbon sources, while urea, beef extract, and peptone were chosen as the most suitable nitrogen sources. Moreover, MnCl_2_ and MgSO_4_ were the metal ions that most strongly stimulated enzyme synthesis. These factors exerted a considerable impact on laccase biosynthesis by the isolated strain and hence were considered for optimization using a Plackett–Burman Design (PBD). CuSO_4_ was added to each flask at a concentration of 0.01 mM by default, along with other factors. The significance of seven factors, comprising various carbon and nitrogen sources and metal ions, was evaluated in a 12-experiment design at two concentration levels. Experiments were performed in statically operated submerged fermentation at pH 9 and 37 °C for 3 days. High and low concentrations for each factor are mentioned in the methodology. Experimental data and their corresponding effects, standard errors, t-ratios, *p*-values, and confidence levels are shown in Tables 2 and 3. According to the statistical analysis, cellulose, urea, and MgSO₄ played significant roles in laccase synthesis, with each having a confidence level above 98%, making them the most important parameters to optimize.

**Table 3:** Statistical analysis of components for laccase production by *Proteus mirabilis* JB1

Components	Effect	t value	*p* Value	% Significance
Cellulose	0.2966667	4.94	0.007817	99.21832
Sucrose	−0.11	1.83	0.141228	85.87724
Urea	0.41	6.83	0.002403	99.75966
Beef E	0.0866667	1.44	0.223287	77.67128
Peptone	−0.0866667	1.44	0.223287	77.67128
MnCl_2_	0.1833333	3.06	0.037658	96.23415
MgSO_4_	0.3766667	6.28	0.003283	99.67172

### CCD-RSM

PBD was used as a screening step to identify the variables that exerted statistically significant effects on laccase production. The statistical analysis showed significant positive effects for cellulose (effect = 0.2967; *p* = 0.007817), urea (effect = 0.4100; *p* = 0.002403), and MgSO₄ (effect = 0.3767; *p* = 0.003283). MnCl₂ also showed a positive effect but was less significant (*p* = 0.037658). In contrast, sucrose and peptone showed negative effects, although these effects were not statistically significant. A positive effect indicates an increase in the level of the particular variable to achieve the optimum response in the experiment. In contrast, a negative effect indicates a decrease in the variable's level to achieve the highest response in the experiment. Therefore, cellulose, urea, and MgSO₄ were retained for CCD. In contrast, the remaining variables were excluded because their effects did not reach the predefined level of statistical significance or because they contributed less strongly to the response. 20 experiments were carried out to come up with a quadratic model having three independent variables, namely cellulose (A), urea (B), and MgSO₄ (C), at varying concentrations to explore the effect of interactions on the laccase production. The experimental design, along with corresponding predictions and observations, is shown in Table 4.

**Table 4:** The experimental CCD with actual and coded levels of variables and the response function

		Factor 1		Factor 2		Factor 3		Response 1	
Std	Run	A: Cellulose		B: Urea		C: MgSO4		Observed	Predicted
		gm %	Coded	gm %	Coded	mM	Coded	U/ml	U/ml
1	19	0.5	−1	0.7	−1	0.05	−1	1.94	1.94
2	17	1.5	1	0.7	−1	0.05	−1	2.23	2.23
3	14	0.5	−1	2.5	1	0.05	−1	1.94	1.81
4	16	1.5	1	2.5	1	0.05	−1	2.08	2.08
5	15	0.5	−1	0.7	−1	0.15	1	1.97	1.97
6	12	1.5	1	0.7	−1	0.15	1	2.03	2.03
7	18	0.5	−1	2.5	1	0.15	1	1.71	1.71
8	5	1.5	1	2.5	1	0.15	1	1.97	1.97
9	9	0.159104	-α	1.6	0	0.1	0	2.03	2.03
10	8	1.8409	+ α	1.6	0	0.1	0	2.3	2.3
11	11	1	0	0.086387	-α	0.1	0	2.08	2.08
12	13	1	0	3.11361	+ α	0.1	0	1.64	1.64
13	10	1	0	1.6	0	0.01591	-α	1.73	1.85
14	7	1	0	1.6	0	0.18409	+ α	1.86	1.86
15	1	1	0	1.6	0	0.1	0	1.87	1.87
16	3	1	0	1.6	0	0.1	0	1.86	1.86
17	6	1	0	1.6	0	0.1	0	1.88	1.88
18	4	1	0	1.6	0	0.1	0	1.88	1.88
19	2	1	0	1.6	0	0.1	0	1.87	1.87
20	20	1	0	1.6	0	0.1	0	1.87	1.7

The production of the laccase enzyme can best be modeled using the following model:

$$\begin{array}{l}Y=1.85+\left({0.0977}^\ast A\right)-\left({0.0981}^\ast B\right)-\\\left({0.0488}^\ast C\right)+\left({0.0225}^\ast A^\ast B\right)\\-\left({0.03}^\ast A^\ast C\right)-\left({0.005}^\ast B^\ast C\right)+\left({0.1087}^\ast A^2\right)\\+\left({0.0009}^\ast B^2\right)+\left({0.0097}^\ast C^2\right)\end{array}$$

in which Y = production of the laccase enzyme (U/ml), A = Cellulose (g%), B = Urea (g%), and C = MgSO_4_(mM).

The model F-value of 21.13 indicates statistical significance, as the probability of obtaining that value by chance alone is only 0.01%. The p-values for the terms A, B, C, and A^2^, all less than 0.05, indicate significance for the model factors, suggesting that linear and quadratic cellulose are vital to enzyme production. The F value for the lack-of-fit term (3.58) was not significant (*p* > 0.05), indicating that the model fits the experimental data well, as represented in Table 5. In addition, the model fits the experimental data well, with a high coefficient of determination (R^2^) and a low CV.

**Table 5:** Analysis of variance for the fitted quadratic polynomial model for level optimization of laccase production using *Proteus mirabilis* JB1

Source	Sum of Squares	df	Mean Square	F-value	p-value	
Model	0.4775	9	0.0531	21.13	< 0.0001	significant
A-Cellulose	0.1303	1	0.1303	51.89	< 0.0001	significant
B-Urea	0.1315	1	0.1315	52.35	< 0.0001	significant
C-MgSO4	0.0325	1	0.0325	12.93	< 0.0049	significant
AB	0.004	1	0.004	1.61	0.2329	
AC	0.0072	1	0.0072	2.87	0.1213	
BC	0.0002	1	0.0002	0.0796	0.7835	
A^2^	0.1704	1	0.1704	67.85	< 0.0001	significant
B^2^	0	1	0	0.0047	0.9468	
C^2^	0.0014	1	0.0014	0.5447	0.4775	
Residual	0.0251	10	0.0025			
Lack of Fit	0.0196	5	0.0039	3.58	0.0939	not significant
Pure Error	0.0055	5	0.0011			
Cor Total	0.5027	19				

**Fig. 2:**
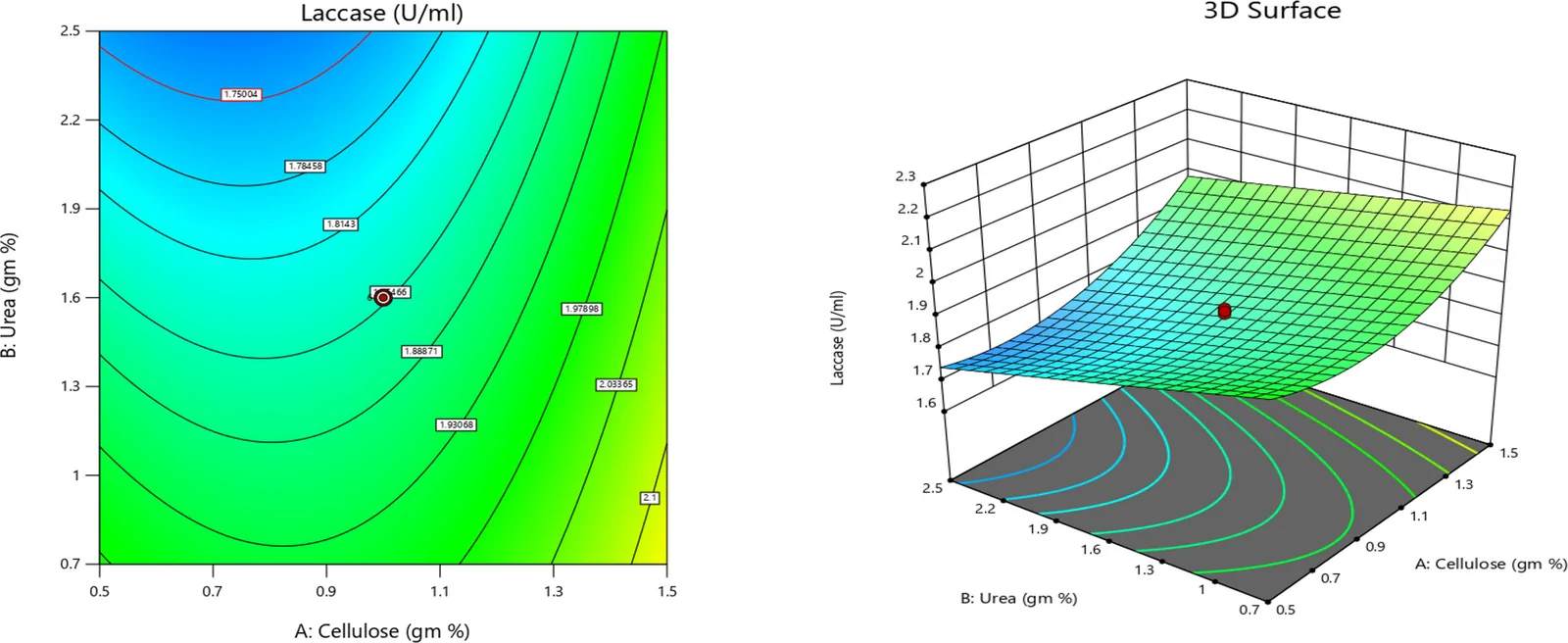
Response surface graph showing the interaction effect of cellulose and urea on laccase production

**Fig. 3:**
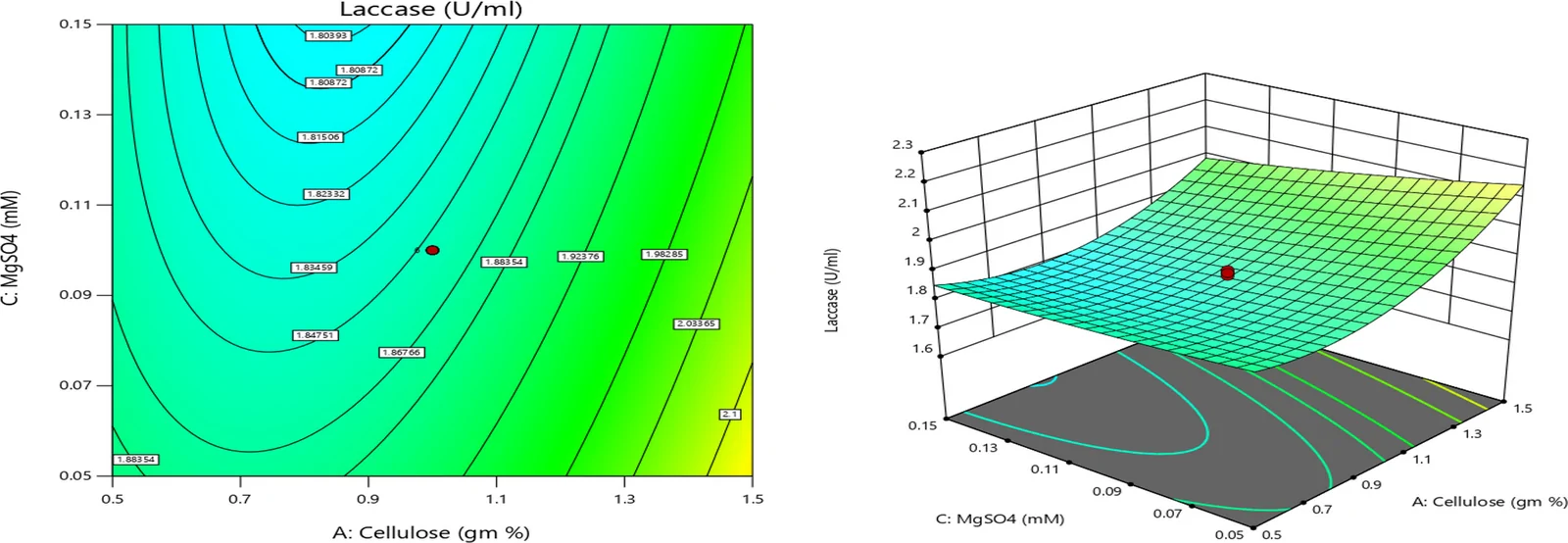
Response surface graph showing the interaction effect of cellulose and MgSO_4_ on laccase production

**Fig. 4:**
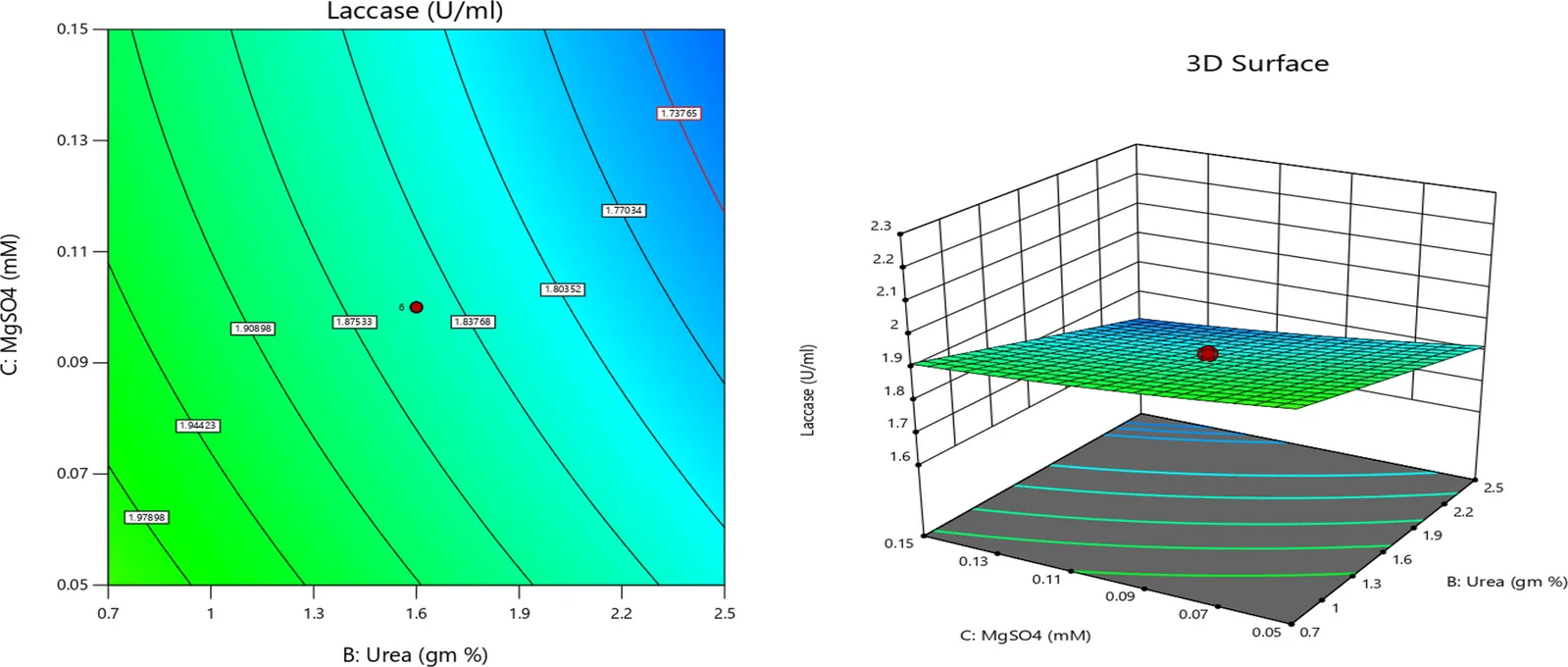
Response surface graph showing the interaction effect of urea and MgSO_4_ on laccase production

Response surface plots and contour graphs were generated to examine parameter interactions and identify optimal points. The interaction between cellulose and urea (Fig. 2) showed that high cellulose concentrations increased laccase production, whereas high urea concentrations inhibited it. The interaction between cellulose and MgSO₄ indicated that higher concentrations of cellulose coupled with lower concentrations of MgSO₄ increased enzyme production, while high MgSO₄ concentrations decreased enzyme production. This is due to interference from high concentrations of inorganic ions, which can cause an osmotic imbalance. As shown in Fig. 3, maximum laccase activity was achieved at higher cellulose concentrations with reduced MgSO₄ levels. In contrast, elevated MgSO₄ combined with high cellulose suppressed enzyme yield, indicating the need for balanced nutrient–ion conditions for optimal biosynthesis. The interaction between urea and MgSO₄ (Fig. 4) also showed that low concentrations of the two variables favored laccase production.

This study makes it clear that laccase production requires a more complex interaction between nutrients and the environment. Synergistic effects observed with high cellulose and low nitrogen concentrations suggest a shift toward secondary metabolism for enzyme production. Antagonistic effects observed with higher salt concentrations may indicate that the cell is under stress.

### Validation of the quadratic model

Based on the ANOVA results, the quadratic model showed a high coefficient of determination (R^2^ ≈ = 0.950), indicating that approximately 95% of the variability in laccase production was explained by the model. The adjusted R^2^ was approximately 0.905, indicating that the model retained a high explanatory power after accounting for the number of model terms. The overall model was highly significant (F = 21.13, *p* < 0.0001), while the lack-of-fit was not significant (F = 3.58, *p* = 0.0939), indicating no statistically significant evidence of inadequate model fit. The predicted R^2^ obtained from Design-Expert software has also been included in the revised manuscript (0.67). The agreement among R^2^, adjusted R^2^, and predicted R^2^ was used to assess the model's predictive reliability.

Experimental validation was subsequently performed under the predicted optimum conditions. The experimentally obtained laccase activity was 2.300 U/mL, compared with a model-predicted value of 2.218 U/mL, demonstrating close agreement between the predicted and experimental values.

The sequential optimization strategy significantly enhanced the laccase activity. Starting from a baseline of 0.240 U/mL in LB medium, activity was improved to 0.999 U/mL (a 4.16-fold increase) using an OFAT approach. PBD subsequently identified cellulose, urea, and MgSO₄ as the critical variables for further optimization. CCD-RSM then accounted for the combined effects and nonlinear relationships among these variables, thereby increasing experimentally validated laccase production to 2.300 U/mL. The comparison demonstrates that the sequential strategy progressively improved laccase production. The initial production level in LB medium was 0.240 U/mL. OFAT optimization increased the activity to 0.999 U/mL (Table 6a). This comparison demonstrates the advantage of combining conventional OFAT optimization with statistical experimental design. OFAT was useful for identifying suitable individual factors. In contrast, PBD efficiently screened the critical variables, and CCD enabled their combined optimization, resulting in an improvement in enzyme activity from 4.16-fold to 9.58 (Table 6b).

**Table 6:** A Optimum Laccase activity after OFAT analysis. b. Enhanced activity of laccase enzyme under statistically optimized parameters

Parameter	OFAT optimum	Laccase activity
Basal medium	LB	0.240 ± 0.001 U/mL
Carbon source (cellulose)	01.00%	0.638 ± 0.004 U/mL
Cellulose concentration	0.25%	0.705 ± 0.0005 U/mL
Nitrogen source (Urea concentration)	01.00%	0.792 ± 0.005U/mL
Urea concentration	0.80%	0.798 ± 0.005 U/mL
pH	9	0.963 ± 0.0005 U/mL
MnCl₂	0.1 mM	0.999 ± 0.003 U/mL
MgSO₄	0.1 mM	0.981 ± 0.005 U/mL
Stage	**Activity (U/mL)**	**Improvement vs. initial**
Initial LB medium	0.240	1.00-fold
OFAT – cellulose	0.705	2.94-fold
OFAT – final combination	0.999	4.16-fold
PBD	Screening stage	—
CCD predicted	2.218	9.24-fold
CCD validated	**2.3**	**9.58-fold**

### Characterization of Lac@SeNPs

The formation of Lac@SeNPs was characterized using the required analytical techniques, including UV–Vis spectroscopy, FTIR spectroscopy, Dynamic Light Scattering (DLS), and Zeta potential**.** A distinct visual transformation in the reaction mixture provided preliminary evidence of laccase-mediated biosynthesis of selenium nanoparticles (SeNPs). The initially colorless sodium selenite solution developed a characteristic ruby-red color after 24 h of incubation (Figure S4), a well-established indicator of SeNP formation. UV further substantiated the formation of SeNPs; visible spectroscopic analysis revealed a pronounced absorption peak at 300–374 nm (Figure S5). This absorption maximum (λ_max) falls within the previously reported range for biologically synthesized SeNPs (240–560 nm), thereby validating the successful formation of nanoparticles in the present system (Ahamad Tarmizi et al. 2023).

To elucidate the molecular interactions underlying nanoparticle synthesis and stabilization, Fourier-transform infrared (FTIR) spectroscopy was employed to compare the spectra of native laccase and the synthesized Lac@SeNPs (Figure S6). The FTIR spectrum of laccase exhibited prominent bands at 430, 451, 1037, 1642, 2103, and 3368 cm⁻^1^. The peaks at 430 and 451 cm⁻^1^ correspond to Cu–O/Cu–N vibrations, indicative of metal–ligand interaction and confirming the multicopper nature of the laccase enzyme. The glycosidic linkage observed at 1037.45, which corresponds to C-O/C-N stretching, confirms the glycoprotein nature of the enzyme. The amide I band at 1642.03 cm⁻^1^ corresponds to C = O stretching of peptide bonds and reflects the secondary structural elements of proteins, including α-helices and β-sheets. The broad band at 3368.06 cm⁻^1^ is attributed to O–H and N–H stretching vibrations, suggesting extensive hydrogen bonding. Minor peaks in the range of 2103 cm⁻^1^ may be assigned to C≡C stretching or interactions involving metal-associated organic moieties.

Following laccase-coated nanoparticle formation, significant spectral modifications were observed in the FTIR profile of Lac@SeNPs. Characteristic peaks were detected at 444.49, 1638.83, 2105.38, and 3439.94 cm⁻^1^. The metal-shift O (444.49 Cm-1) vibration indicates a strong interaction between selenium and a proteinaceous environment. The persistence of the amide I band at 1638.83 cm⁻^1^ indicates the continued association of proteinaceous components with the nanoparticle surface, highlighting their role in capping and stabilizing the nanoparticle. The disappearance of the C–O stretching band at 1037.45 cm⁻^1^ suggests the involvement of glycosidic moieties in the reduction process, possibly through structural modification or cleavage. The emergence of a distinct peak at 2105.38 cm⁻^1^ indicates the formation of selenium–organic interactions, likely due to selenium binding to functional groups within the enzyme matrix. Furthermore, the slight shift in the broad O–H/N–H stretching band reflects changes in hydrogen-bonding patterns, indicative of conformational rearrangements during nanoparticle synthesis. The DLS results of the synthesized laccase-coated selenium nanoparticles (Lac@SeNPs) showed a single distinct peak corresponding to an average hydrodynamic diameter of 376.7 nm with a polydispersity index (PDI) of 0.206. The lower PDI value indicates a narrower size distribution, thereby indicating the formation of a homogeneous dispersion of nanoparticles. Also, the measured zeta potential of − 20.73 mV suggests moderate stability of the colloid (Figure S7). The immobilization efficiency was determined by quantifying the residual protein concentration in the supernatant following enzyme conjugation to the nanoparticles. The initial protein concentration was 860 ± 0.005 µg/ml, which decreased to 218 ± 0.041 µg/ml post-immobilization, indicating that 642 ± 0.001 µg/ml of protein was successfully conjugated. This corresponds to an immobilization efficiency of 74.65%. This reduction in soluble protein confirms successful enzyme surface coating (Table 7).

**Table 7:** Quantification of Immobilization Efficiency

Parameter analyzed	Concentration
Initial Protein	860 ± 0.005 μg/ml
Protein present in the supernatant	218 ± 0.041 μg/ml
Total immobilized protein	642 ± 0.001 μg/ml
Immobilization yield	74.65%

### Cypermethrin detoxification assay

Cypermethrin was identified using UV–Visible spectroscopy. The compound exhibited an absorption maximum (λmax) at approximately 268 nm, attributable to aromatic conjugation (Figure S8). In the course of detoxification of this compound, there was a decrease in absorbance at λmax in all concentrations ranging from 0.3 mM to 1.0 mM, as presented in Figure S9. Among these samples, the 1 mM sample was analyzed by FTIR to assess structural modifications of cypermethrin upon interaction with various biocatalytic systems, as shown in Table S3a. The untreated control sample (C; cypermethrin) displayed typical absorption peaks for ~ 3369 cm⁻^1^ (O–H/N–H), 2091 cm⁻^1^ (C≡X stretching), 1640 cm⁻^1^ (C = C/C = O), and halogen peaks at 413–746 cm⁻^1^, confirming its aromatic and halogenated structure. During treatment, the samples exhibited gradual spectral changes, indicating stepwise detoxification of the pesticide. The B sample (B: Direct inoculation of *Proteus mirabilis* JB1 strain) showed the absence of prominent aromatic ring peaks with minimal shifts in halogen peaks, denoting the breakdown of aromatic rings and partial conversion. In the case of E (E: Partially purified laccase enzyme), the presence of broadened peaks in the halogen region, alongside retention of the C = C band, indicates moderate detoxification with partial structural preservation. However, the most drastic change in peak intensity was observed in the Se + BE (Se + BE: Lac@SeNPs) sample, wherein the peak for C≡X was absent, along with broadened peaks in the metal interaction. These findings suggest that enzyme-coated nanoparticles are the best option for such applications, as they provide greater stability and sustainability for the enzyme (Figure S10).

### Confirmation of cypermethrin detoxification by GC–MS and its metabolites profiling

GC MS further confirmed cypermethrin detoxification (Table S3b). In the control test, the cypermethrin peak was detected at 25.0- 25.3 min, with an area of 3,521,090, corresponding to 100% residual pesticide. On the contrary, no cypermethrin peak was detected in any of the treated tests with B, E, and Se + BE, indicating complete degradation, with 100% efficiency. This finding provides robust corroboration with FTIR and shows complete detoxification of cypermethrin. Metabolites obtained from GC–MS analysis include: Long-chain hydrocarbons (heneicosane, eicosane, tetratriacontane), Oxygenated intermediates (ethyl acetate derivatives), Phenols (2,4-di-tert-butylphenol), and alkane derivatives. From this observation, it can be concluded that cypermethrin detoxification is associated with oxidation of ester linkages, aromatic ring fragmentation, and further conversion to simpler aliphatic/phenolic intermediates. The GC chromatogram is shown in Figure S11. It is important to note that the Se + BE treatment system yielded balanced intermediates, suggesting an efficient detoxification pathway.

## Discussion

Industrial effluents and effluent-contaminated soil are ideal niches for extremophilic microorganisms due to their harsh physicochemical conditions; such environments are promising sources for bioprospecting novel extremozyme-producing microorganisms. Microbial strains adapted to such chemical stress are physiologically capable of producing pollutant-degrading enzymes such as laccases (Chandel et al. 2026). Primary screening includes plate assays to quickly assess isolates' ability to secrete laccase enzymes, as indicated by reddish-brown coloration surrounding the colonies on guaiacol, a model substrate for lignin-degrading enzymes such as laccases and peroxidases (Mehandia et al. 2020). Secondary screening involves submerged fermentation and spectrophotometric assays to measure enzyme production, activity, and stability in greater detail (Wiśniewska et al. 2021). This systematic approach selects the most promising candidates for further optimization. Variation in laccase activity among isolated strains reveals the metabolic diversity inherent in bacterial communities from diverse environmental sources. Previous reports highlight the suitability of rapid screening to identify potential isolates for further research (Kumar et al. 2025).

Initial screening of media components was conducted using the One-Factor-at-a-Time (OFAT) method to identify key parameters influencing laccase production (Deepa et al. 2020; Thomas et al. 2025). The initial concentration ranges were selected based on commonly used concentrations reported for microbial enzyme production and preliminary laboratory observations. Carbon and nitrogen sources were initially screened at standardized concentrations to enable comparison among substrates, followed by concentration optimization of the most promising sources. Similarly, metal ions were initially evaluated at 0.01 and 0.1 mM to identify both stimulatory and inhibitory effects while avoiding excessive ionic concentrations. Optimizing laccase synthesis via the OFAT technique revealed that laccase production was significantly affected by both nutritional and physicochemical factors. The present study showed that cellulose induced the highest activity among the tested carbon sources. This may be attributed to its ability to provide a sustained carbon source and to stimulate secondary metabolic pathways associated with extracellular oxidative enzymes. In contrast, readily metabolizable soluble sugars may preferentially support biomass formation and may exert carbon catabolite effects that reduce enzyme production. This suggests that, in the presence of an inducer such as guaiacol, complex carbon sources can more effectively promote growth and enzyme formation than readily degradable carbon sources (Mahuri et al. 2023). The increased enzyme activity in the presence of guaiacol on complex carbon sources can be explained by the substrate's dual nature: it serves not only as a nutrient but also as an inducer. Guaiacol, a representative aromatic molecule, serves as a diagnostic substrate that induces laccase formation by stabilizing the enzyme's copper-binding active site. The mechanisms of induction described above align with the ecological roles of fungi and bacteria as lignocellulose degraders, in which aromatic compounds function as inducers of enzymatic reactions (Shraddha et al. 2011). One important factor is the availability of organic nitrogen sources, which are essential for enzyme production and provide amino acids, vitamins, and growth factors. The previous report also supports recent findings that indicate a major role for nitrogen in enhancing laccase production (Colla et al. 2023). For the nitrogen source, the observation aligns with previous reports showing that complex organic nutrients stimulate the biosynthesis of secondary metabolites and enzymes in fungi and bacteria (Parshad et al. 2025; Sun et al. 2024). Among the nitrogen sources, a favorable response to urea may reflect efficient nitrogen assimilation while avoiding excessive availability of organic nitrogen, which could favor biomass formation over extracellular enzyme synthesis. Excess nitrogen appeared to repress enzyme synthesis, possibly via catabolite repression mechanisms, in which high nutrient availability diverts metabolic flux toward primary metabolism rather than secondary enzyme production (Cunningham et al. 2000). Unlike most reported laccases, which typically show high activity at acidic to neutral pH and low stability at alkaline pH (Benavides et al. 2024), JB1 exhibits stability at an alkaline pH of 11, distinguishing it from the majority of reported laccase-producing strains. Such extremozymes have substantial direct applications in all the remedial and biotechnological applications performed under alkaline conditions. The favorable response at alkaline pH may reflect the isolate's adaptation to alkaline environmental conditions and the maintenance of cellular processes associated with enzyme secretion under elevated pH. Since laccase production is influenced by nutrient transport, cellular metabolism, and extracellular enzyme stability, an appropriate alkaline pH may provide a favorable physiological environment for enzyme biosynthesis and secretion. Metal ions play a major role in laccase function, as previously reported. Also, previous reports supported the present findings of the influence of copper, manganese, and magnesium ions on laccase activity (Benavides et al. 2024; Stajic et al. 2006). The enhanced response to MnCl₂ and other selected mineral ions may be associated with their roles in cellular metabolism, enzyme synthesis, metal homeostasis, and maintenance of the metalloenzyme system. However, the effect was concentration-dependent, indicating that metal ions may stimulate enzyme production at low concentrations but become inhibitory at higher concentrations.

Surfactants can affect enzyme activity in different ways. Ionic surfactants typically reduce enzyme activity, likely by disrupting hydrophobic interactions and destabilizing protein folding. On the other hand, non-ionic surfactants often have little effect on activity, and sometimes even help dissolve substrates without harming the enzyme’s structure (Liu et al. 2025). Inhibitors such as metal chelators often lower enzyme activity by sequestering essential metal ions, thereby impairing catalytic efficiency. In laccases, these inhibition patterns show that the enzyme depends on metal cofactors, especially copper (Cu), which is needed for its catalytic function (Elsayed et al. 2023). Organic solvents affect enzymes differently depending on their concentration. At low amounts, they can help substrates dissolve and make enzymes more accessible. At higher levels, they can stop enzyme activity by causing proteins to lose their structure and damaging membranes. Some microbes can tolerate polar solvents, which suggests their enzymes are either more stable or supported by cofactors (Jafari et al. 2020). The selected variables comprised cellulose and sucrose as carbon-related factors; urea, beef extract, and peptone as nitrogen-related factors; and MnCl₂ and MgSO₄ as mineral/metal factors. These variables were selected because they showed measurable effects on laccase production during preliminary OFAT screening and therefore warranted simultaneous statistical evaluation. PBD was used specifically as a screening design to distinguish variables with statistically meaningful effects from those with comparatively small effects. Cellulose, urea, and MgSO₄ showed the strongest positive effects and high confidence levels and were therefore selected for subsequent CCD optimization.

PBD and CCD analyses from earlier investigations have shown that glucose, yeast extract, and metal ions such as copper and iron significantly stimulate laccase formation in *Enterobacter* species (Hosseini et al. 2025). It is well known that certain inorganic compounds play important roles in laccase production and secretion. Notably, NH₄Cl, MnSO₄, and CuSO₄ have a stimulatory effect on laccase production (Abdelgalil et al. 2022). The present study identifies cellulose, urea, and MgSO_4_ as potential media components. These results corroborate earlier studies showing that, in addition to serving as a substrate, more complex carbon sources, such as cellulose, act as inducers, stimulating laccase synthesis via ligninolytic pathways (Elisashvili et al. 2008; Piscitelli et al. 2011). In other words, the increase in enzyme yield at higher cellulose concentrations could be linked to upregulation of laccase-encoding genes, induced by oxidative enzymes that degrade recalcitrant polymers (Janusz et al. 2020). Induction, in turn, is usually mediated by transcriptional regulators responding to lignocellulosic substrates (Mate and Alcalde 2015). Cellulose may act not only as a carbon source but also as a relatively slowly metabolized substrate, thereby supporting sustained carbon availability. In contrast to readily assimilable soluble sugars, slower carbon release may reduce excessive biomass-directed metabolism and favor the expression of extracellular oxidative enzymes associated with secondary metabolism. The observed increase in laccase production in the presence of cellulose is therefore likely related to the balance among carbon availability, growth, and the synthesis of secondary metabolic enzymes. The subsequent PBD analysis confirmed cellulose as a statistically significant factor, supporting its importance in laccase biosynthesis.

On the contrary, increased nitrogen concentration appears to reduce the efficiency of laccase synthesis, most likely via nitrogen catabolic repression (NCR), a regulatory pathway used by many microorganisms to repress secondary metabolism, particularly ligninolytic enzymes, when nitrogen is abundant (Baldrian 2006). In line with the theory described above, the decreased enzyme yield efficiency observed as urea concentration increases can be explained within the NCR framework. During OFAT, MgSO₄ was evaluated individually while the other medium components were maintained at fixed concentrations. Under these conditions, 0.1 mM MgSO₄ supported high laccase activity (0.981 U/mL), indicating that MgSO₄ is beneficial when evaluated as an individual factor. In contrast, CCD simultaneously evaluated MgSO₄ in the presence of cellulose and urea, thereby capturing interactions and nonlinear effects that OFAT cannot detect. The response surface analysis demonstrated that higher cellulose concentrations combined with lower MgSO₄ concentrations favored laccase production, whereas elevated MgSO₄ concentrations reduced the response. Thus, the apparent difference does not represent a contradiction; rather, it demonstrates the limitation of single-factor optimization and the advantage of response surface methodology. Moreover, the effects of MgSO₄ provide insights into the role of mineral ions in regulating enzyme production. Although metal ions are important for the structural stability and function of cells and enzymes, their levels should be carefully controlled. While an appropriate amount of magnesium ions (Mg^2^⁺) can enhance enzyme stability and metabolism, higher concentrations can disrupt the balance among ionic concentration, osmotic pressure, and protein folding or secretion. This phenomenon has been previously observed, with metal ions activating or inhibiting reactions depending on concentration and the cellular systems involved (Najafabadipour et al. 2021). CCD was subsequently used because, unlike PBD, it permits evaluation of nonlinear effects and interactions among the selected variables. Thus, PBD served as the variable-screening stage, whereas CCD served as the response-surface optimization stage.

Despite the growing interest in enzyme-assisted nanoparticle synthesis, the application of laccase for this purpose remains largely unexplored. In this context, the present study provides novel evidence for the utilization of laccase in the green synthesis of SeNPs. Enzymes facilitate the reduction of metal ions by donating electrons, thereby forming zero-valent nanoparticles. Additionally, biomolecular functional groups stabilize nanoparticles through electrostatic interactions and surface binding (Pasinszki and Krebsz 2020). The diameters have been similar to those previously recorded for other biogenic or protein-coated selenium nanoparticles (Zhang et al. 2024). Despite the size of the synthesized particles being higher than that of the bare selenium nanoparticles (50–200 nm) (Geng et al. 2025), this is a usual trend that has been observed when an enzyme or protein is used for coating, owing to surface adsorption of proteins, hydration layer formation, and interaction between the coated particles. The zeta potential value is attributed to the ionizable groups (amino acids) of the adsorbed laccase molecule at the nanoparticle surface. According to previous reports, the zeta potential values for selenium nanoparticles capped with proteins range between − 15 and − 35 mV (Tugarova et al. 2018). The depletion of protein concentration implies that the resulting nanoparticles exhibit good dispersion stability and successful enzyme functionalization. The results provide sufficient evidence that stable laccase-coated selenium nanoparticles (Lac@SeNPs) were formed and that laccase is an effective stabilizing and capping agent for their synthesis. SeNPs provide a greater surface area for enzyme immobilization, reduce enzyme denaturation, and increase the biocatalyst's thermal and pH stability and reusability. Thus, the Lac-SeNP system is a sustainable alternative for remediating highly contaminated environments. Previous studies have shown that no strains have the potential to degrade 100% of cypermethrin within a short period of time (72 h) (Bhatt et al. 2020; Das et al. 2022; Huang et al. 2018).

The enhanced catalytic performance of Lac@SeNPs may result from the nanoscale surface providing a high-surface-area interface for enzyme association and from interactions between laccase functional groups and the selenium nanoparticle surface. Such immobilization can help maintain the enzyme's active conformation and reduce deactivation under environmental conditions. The nanoparticle interface may also improve the dispersion of the biocatalyst in the reaction medium and increase the effective contact between the enzyme and hydrophobic pesticide molecules, such as cypermethrin. The FTIR results support the interaction between protein functional groups and the selenium nanoparticle surface. The DLS analysis showed an average hydrodynamic diameter of 376.7 nm with a PDI of 0.206. At the same time, the zeta potential was − 20.73 mV, supporting the formation of a relatively stable enzyme-associated nanoparticle system.

Compared with previous microbial and immobilization systems, the present study, focusing on Proteus mirabilis JB1 with Lac@SeNP (laccase–selenium nanoparticle conjugates), represents a significant advancement in cypermethrin detoxification. *Bacillus cereus* GW-01 ΔcodY mutants improved degradation by reducing lag phases and accelerating aromatic clearance (Lei et al. 2027), while *Bacillus thuringiensis* SG4 achieved 83–85% removal in both slurry and immobilized matrices (Bhatt et al. 2020). In contrast, the current study achieved complete removal of the parent compound within 72 h at a concentration of 1 mM. GC–MS analysis confirmed the absence of cypermethrin and identified downstream metabolites, including Dodecane (4,6-dimethyl-), 2,4-Di-tert-butylphenol, and Heneicosane, indicating full conversion rather than partial transformation Table S4. These results highlight the practical potential of nanobiocatalytic approaches to complement genetic and immobilization strategies, enabling rapid clearance of hydrophobic pesticide residues and preventing the buildup of toxic intermediates such as 3-phenoxybenzoic acid. While not claiming universal superiority, these findings suggest that nanoparticle–enzyme conjugation is a valuable addition to the bioremediation toolkit, especially when complete removal of the parent compound is environmentally beneficial.

GC–MS analysis identified metabolites such as Dodecane (4,6-dimethyl-), 2,4-Di-tert-butylphenol, and Heneicosane, indicating that cypermethrin was fully degraded rather than partially transformed. These results suggest a stepwise process: laccase first cleaves the ester bond, producing short-lived intermediates such as 3-phenoxybenzaldehyde and 3-phenoxybenzoic acid. These aromatic intermediates are then oxidized and destabilized, resulting in ring opening and conversion to smaller aliphatic hydrocarbons. The presence of long-chain hydrocarbons demonstrates that the hydrophobic portions of cypermethrin are ultimately broken down into less toxic, biodegradable compounds.

**Fig. 5:**
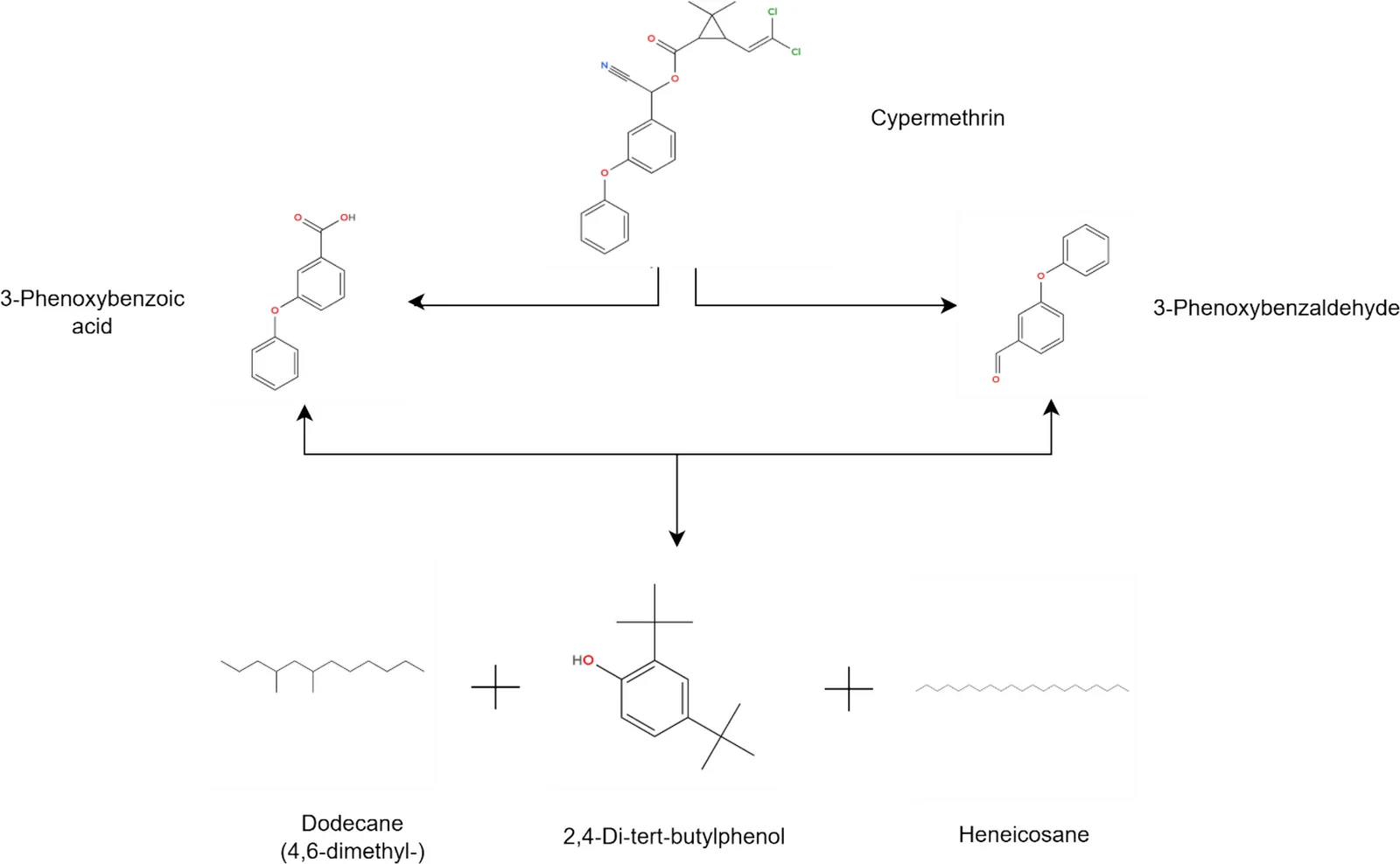
Pathway for cypermethrin degradation by Lac@SeNPs

This proposed pathway is consistent with established enzymatic mechanisms of pyrethroid degradation, in which esterases and laccases jointly cleave ester bonds and oxidize aromatic rings. In our system, immobilizing laccase on selenium nanoparticles likely enhanced catalyst stability and electron transfer, accelerating the breakdown. The complete disappearance of cypermethrin after 72 h confirms that detoxification was achieved through full conversion (Fig. 5).

## Conclusion

A recent study introduces a novel technique for decontaminating cypermethrin using a nanobiocatalyst containing extremophilic enzymes. The laccase-producing bacterial strain *Proteus mirabilis* JB1, isolated from an extreme environment, demonstrated stable enzyme activity at alkaline pH up to 9 and is therefore considered a promising and novel source of robust enzymes for future applications.

A systematic, sequential statistical optimization strategy, from traditional OFAT analysis to PBD and CCD-RSM, resulted in a 9.58-fold increase in enzyme activity. Enzyme production in the selected microorganism was improved through systematic optimization. Initial screenings in minimal salt and Luria–Bertani media yielded enzyme activities of 0.220 U/mL and 0.240 U/mL, respectively. The Plackett–Burman Design identified cellulose (1.5% w/v), urea (0.7% w/v), and MgSO₄ (0.05 mM) as the most significant factors for enzyme production. Central Composite Design led to an enhanced enzyme activity of 2.300 U/mL.

The biogenic immobilization of this extremozyme-based laccase with selenium nanoparticles (Lac@SeNPs) facilitated an efficient catalytic remediation of cypermethrin. This suggests the potential synergism between the enzymatic activity and the functional properties of SeNPs. GC–MS analysis demonstrated the effective structural transformation of the parent cypermethrin molecule; however, given the complexity of pesticide metabolites, we recognize the need for further ecotoxicity and phytotoxicity evaluations to confirm the safety of the transformation. In summary, this research bridges the gap between enzyme engineering and nanobiotechnology, providing a robust, replicable framework for the development of sustainable, reusable biocatalytic platforms. While our laboratory-scale results indicate substantial potential to mitigate pesticide residues, future studies should focus on evaluating the scalability and long-term operational stability of the Lac@SeNPs system under complex, field-scale conditions.

In summary, this study bridges enzyme engineering and nanobiotechnology, offering an advanced solution for efficient, sustainable pesticide removal.

## Supplementary Information

Below is the link to the electronic supplementary material.

ESM 1 (DOCX 1.70 MB)

## Data Availability

No datasets were generated or analysed during the current study.
