# Supplementary material for: Next-generation nano-biocatalysts: extremozyme-based nanoparticle system for cypermethrin bioremediation
Source: Appl Microbiol Biotechnol. 2026 Oct 17;110:321. doi: 10.1007/s00253-026-14053-z (PMC13719933; doi:10.1007/s00253-026-14053-z)
Supplement: Supplementary file 1 [file 253_2026_14053_MOESM1_ESM.docx]

Table S1 Experimental range and level of the independent variables of selected components for RSM – CCD

| Variable Code | Variable | +a | +1 | 0 | -1 | -a |
| --- | --- | --- | --- | --- | --- | --- |
| A | Urea | 3.11361 | 2.5 | 1.6 | 0.7 | 0.0863865 |
| B | Cellulose | 1.8409 | 1.5 | 1 | 0.5 | 0.159104 |
| C | MgSO_4_ | 0.18409 | 0.15 | 0.1 | 0.05 | 0.0159104 |

Table S2 Secondary Screening and Selection of Potential Isolates Based on Laccase Activity

| **Isolates** | **pH** | **Enzyme Activity at pH 7 (U/ml)** | **Enzyme Activity at pH 9 (U/ml)** |
| --- | --- | --- | --- |
| JA1 | 6 and 7 | 0.015 | 0.005 |
| JA2 | 6 and 7 | 0.011 | 0.023 |
| JA3 | 6 and 7 | 0.073 | 0.063 |
| JA5 | 6 and 7 | 0.086 | 0.075 |
| JA6 | 6 and 7 | 0.098 | 0.078 |
| JA7 | 6 and 7 | 0.011 | 0.001 |
| **JB1** | **7 and 9** | **0.156** | **0.240** |
| JB2 | 7 and 9 | 0.080 | 0.042 |
| JB3 | 7 and 9 | 0.015 | 0.166 |
| JB4 | 7 and 9 | 0.009 | 0.012 |
| JB5 | 7 and 9 | 0.007 | 0.080 |
| JB6 | 7 and 9 | 0.153 | 0.226 |

Table S3a. Quantification of Cypermethrin detoxification by FTIR analysis

| Sample | ~3300 cm⁻¹ (O–H/N–H) | ~2090 cm⁻¹ (C≡X) | ~1640 cm⁻¹ (C=C/C=O) | 400–500 cm⁻¹ (Halogen/M–O) | Unique/Notable Peaks | Detoxification Inference |
| --- | --- | --- | --- | --- | --- | --- |
| C (Cypermethrin) | 3369.16 | 2091.64 | 1640.20 | 413.59, 746.30 | Strong aromatic ring band | Baseline compound |
| B | 3367.44 | 2099.32 | 1641.20 | 422.07 | No aromatic ring peak | Possible ring modification |
| E | 3369.31 | 2099.24 | 1641.84 | 454.03, 435.07 | Shift in halogen band | Bond cleavage, halogen displacement |
| Se+BE | 3372.89 | - | 1641.20 | 468.17, 439.40, 413.19 | Multiple split peaks | Extensive detoxification & metal interaction |

Table S3b. Quantification of Cypermethrin detoxification by GC

| Sample | Major cypermethrin signal | Major observed by‑products/peaks (RT & approximate area) | Interpretation |
| --- | --- | --- | --- |
| Control (Ethyl acetate) | Cypermethrin peaks at RT 25.045 (1,140,603); 25.197 (873,797); 25.290 (838,751); 25.350 (667,939). Total ≈ 3,521,090 | Heneicosane, Eicosane, Dodecane derivatives | Parent present — baseline. |
| SE+BE | No parent detected (cypermethrin absent) | 2,4‑Di‑tert‑butylphenol (RT ~12.56; area ≈ 563,400), various alkanes (heneicosane, etc.) | Advanced breakdown: oxidative product + hydrocarbons. |
| B | No parent detected | Dominant long‑chain alkanes (several large peaks assigned to heneicosane; large summed area of hydrocarbon peaks) | Parent degraded; chromatogram dominated by matrix hydrocarbons. |
| E | No parent detected | 2,4‑Di‑tert‑butylphenol (RT ~12.56; area ≈ 1,268,733), hexadecane, dodecane derivatives | High phenolic intermediate signal — strong evidence of oxidative cleavage. |

Table S4: Comparative review of bioremediation of cypermethrin

| **Microorganism / System** | **Nanoparticle / Immobilization Type** | **Enzyme Activity / Key Enzyme** | **Detoxification Efficiency** | **Last Products of Detoxification** | **Reference** |
| --- | --- | --- | --- | --- | --- |
| ***Bacillus cereus* GW‑01**  **Wild-Type** | None | Esterase/hydrolase pathway; oxidative stress constrained | 57.76% (soil microcosm, 30 d) | 3‑Phenoxybenzoic acid (3‑PBA), phenol, benzoic acid, catechol derivatives | (Lei et al., 2027) |
| ***B. cereus GW‑01***  **ΔcodY mutant** | None (genetic de‑repression) | Enhanced hydrolase + stress adaptation (↑SOD, biofilm, hydrophobicity) | 63.69% (soil microcosm, 30 d); 76.16% (liquid, 7 d, 50 mg/L) | Same pathway as WT, but faster clearance of 3‑PBA and aromatic intermediates | (Lei et al., 2027) |
| ***B. thuringiensis* SG4** | None | Esterase cleavage of the ester bond | 83.3% (soil slurry, 15 d) | Phenol, 3‑phenoxybenzaldehyde, phthalic acid, 3‑phenoxybenzoate, 2‑hydroxy‑3‑phenoxy‑benzeneacetonitrile | (Bhatt et al., 2020) |
| ***B. thuringiensis* SG4** | Sodium alginate beads | Esterase activity retained | 85.3% (liquid, 15 d, 50 mg/L) | Same metabolite profile as free cells (phenol, phthalic acid, 3‑PBA derivatives) |  |
| ***B. thuringiensis* SG4** | Agar discs | Esterase activity retained | 81.0% (liquid, 15 d, 50 mg/L) | Same metabolite profile as above |  |
| ***Aspergillus niger* YAT** | None | Esterase + co‑metabolism | 54.83% (liquid, 7 d, 50 mg/L) | 3‑Phenoxybenzoic acid → protocatechuic acid → catechol → mineralization | (Huang et al., 2018) |
| ***Aspergillus oryzae* M‑4** | None | Esterase | 26.01% (liquid, 7 d, 50 mg/L) | Aromatic intermediates (3‑PBA, phenolic compounds) |  |
| ***Bacillus licheniformis* B‑1** | None | Esterase | 52.91% (liquid, 7 d, 50 mg/L) | Aromatic intermediates (benzoic acid derivatives) |  |
| ***Proteus mirabilis* JB1** | Lac@SeNP | Laccase enzyme | 100 %, liquid, 72h, 1mM | Dodecane, 4,6-dimethyl-, 2,4-Di-tert-butylphenol, Heneicosane | This manuscript |

Protein Standard Graph

Optical Density at 650nm

| Protein Concentration μg/ml | OD |
| --- | --- |
| 0 | 0 |
| 40 | 0.15 |
| 80 | 0.32 |
| 120 | 0.42 |
| 160 | 0.55 |
| 200 | 0.68 |

Figure S1: Standard curve for protein quantification using BSA

Figure S2: The Phylogenetic tree showing the relationship between isolated *Proteus mirabilis* JB1 (PQ160485) and other closely related sequences on NCBI GenBank.

Figure S3: Effect of various inhibitor, surfactants, and organic solvents

Figure S4: Formation of Lac@SeNp

Figure S5: UV spectra of lac@SeNp

Figure S6: FTIR spectra of lac@SeNp and laccase enzyme

Figure S7: DLS and Zeta potential of Lac@SeNp

Figure S8: UV spectra of cypermethrin.

Cypermethrin Detoxification

Absorbance at 268 nm

| Concentration (mM) | 0 | 12 | 24 | 48 | 72 |
| --- | --- | --- | --- | --- | --- |
| 0.3 B | 0.896 | 0.012 | 0.001 | 0 | 0 |
| 0.3 E | 0.896 | 0.001 | 0 | 0 | 0 |
| 0.3 Se+BE | 0.896 | 0.058 | 0 | 0 | 0 |
| 0.4 B | 1.668 | 0.056 | 0.004 | 0 | 0 |
| 0.4 E | 1.668 | 0.007 | 0 | 0 | 0 |
| 0.4 Se+BE | 1.668 | 0.001 | 0 | 0 | 0 |
| 0.6 B | 2.571 | 0.081 | 0.001 | 0 | 0 |
| 0.6 E | 2.571 | 0.056 | 0.001 | 0 | 0 |
| 0.6 Se+BE | 2.571 | 0.012 | 0.001 | 0 | 0 |
| 0.7 B | 3.56 | 0.194 | 0.008 | 0 | 0 |
| 0.7 E | 3.56 | 0.101 | 0.001 | 0 | 0 |
| 0.7 Se+BE | 3.56 | 0.089 | 0.001 | 0 | 0 |
| 0.9 B | 3.786 | 1.54 | 0.09 | 0 | 0 |
| 0.9 E | 3.786 | 1.2 | 0.007 | 0 | 0 |
| 0.9 Se+BE | 3.786 | 1.07 | 0.004 | 0.001 | 0 |
| 1 B | 3.88 | 1.89 | 0.785 | 0.507 | 0.455 |
| 1 E | 3.88 | 1.056 | 0.65 | 0.425 | 0.12 |
| 1 Se+BE | 3.88 | 1.007 | 0.568 | 0.108 | 0.04 |

Figure S9: Cypermethrin detoxification by bacteria, free enzyme, and immobilized enzyme

Figure S10: FTIR spectra for Control, B, E, Se+BE

Figure S11 GC chromatogram for Control, B, E, Se+BE
